# Geographic variation in hybridization across a reinforcement contact zone of chorus frogs (*Pseudacris*)

**DOI:** 10.1002/ece3.3443

**Published:** 2017-10-11

**Authors:** Emily Moriarty Lemmon, Thomas E. Juenger

**Affiliations:** ^1^ Department of Biological Science Florida State University Tallahassee FL USA; ^2^ Department of Integrative Biology University of Texas, Austin Austin TX USA

**Keywords:** acoustic signal, cascade reinforcement, hybridization, reproductive character displacement

## Abstract

Reinforcement contact zones, which are secondary contact zones where species are diverging in reproductive behaviors due to selection against hybridization, represent natural laboratories for studying speciation‐in‐action. Here, we examined replicate localities across the entire reinforcement contact zone between North American chorus frogs *Pseudacris feriarum* and *P. nigrita* to investigate geographic variation in hybridization frequencies and to assess whether reinforcement may have contributed to increased genetic divergence within species. Previous work indicated these species have undergone reproductive character displacement (RCD) in male acoustic signals and female preferences due to reinforcement. We also examined acoustic signal variation across the contact zone to assess whether signal characteristics reliably predict hybrid index and to elucidate whether the degree of RCD predicts hybridization rate. Using microsatellites, mitochondrial sequences, and acoustic signal information from >1,000 individuals across >50 localities and ten sympatric focal regions, we demonstrate: (1) hybridization occurs and (2) varies substantially across the geographic range of the contact zone, (3) hybridization is asymmetric and in the direction predicted from observed patterns of asymmetric RCD, (4) in one species, genetic distance is higher between conspecific localities where one or both have been reinforced than between nonreinforced localities, after controlling for geographic distance, (5) acoustic signal characters strongly predict hybrid index, and (6) the degree of RCD does not strongly predict admixture levels. By showing that hybridization occurs in all sympatric localities, this study provides the fifth and final line of evidence that reproductive character displacement is due to reinforcement in the chorus frog contact zone. Furthermore, this work suggests that the dual action of cascade reinforcement and partial geographic isolation is promoting genetic diversification within one of the reinforced species.

## 1 INTRODUCTION

Contact zones between recently diverged taxa represent natural laboratories for studying how reinforcement, the process by which selection against hybridization drives an increase in prezygotic isolation (Blair, 1955; Dobzhansky, 1937, 1940; Howard, 1993), leads to the final stages of speciation. One outcome of reinforcement is the pattern of reproductive character displacement (RCD), where reproductive behaviors evolve to be more divergent between species in sympatry than allopatry (Servedio & Noor, 2003; Lemmon, Smadja, & Kirkpatrick, 2004; Nosil, 2012; but see Pfennig & Pfennig, 2009). Selection against hybridization in contact zones is not only thought to contribute to divergence between species but also to promote diversification within species as a result of different selection pressures across allopatric and sympatric populations (Abbott, 2013; Fuller, 2016; Hoskin & Higgie, 2010; Ortiz‐Barrientos, Grealy, & Nosil, 2009; Pfennig & Pfennig, 2009).

Mathematical theory indicates that reinforcement can contribute to the evolution of reproductive isolation under a certain range of conditions, where hybridization occurs at an intermediate rate. If hybridization and/or recombination are too high, the evolution of isolation will be hindered due to homogenization of the interacting taxa (Barton, 2001; Barton & Hewitt, 1989; Britch, Cain, & Howard, 2001; Cain, Andreasen, & Howard, 1999; Kelly & Noor, 1996; Kirkpatrick, 2000; Kirkpatrick & Servedio, 1999; Sanderson, 1989; Servedio, 2000, 2004; Servedio & Kirkpatrick, 1997; Servedio & Noor, 2003). At least a low level of gene flow, however, is required to generate hybrids and provide the opportunity for selection to drive the evolution of reproductive isolation in sympatry (Kirkpatrick, 2000). Thus in nature, the expectation is that observed hybridization rates should be moderate to low in reinforcement contact zones, which are secondary contact zones in which selection against hybridization is driving the evolution of prezygotic isolation between taxa. There is some support for this prediction from empirical data (Sætre et al., 1997; Sætre, Král, Bureš, & Ims, 1999; Nosil, Crespi, & Sandoval, 2003; Borge, Lindroos, Nádvorník, Syvänen, & Sætre, 2005; Hoskin, Higgie, McDonald, & Moritz, 2005; Peterson et al., 2005; Saether et al., 2007; Wiley, Qvarnström, Andersson, Borge, & Sætre, 2009; Matute, 2010; but see Hopkins, Levin, & Rausher, 2012). Another theoretical prediction relates to the directionality of gene flow in contact zones. In exploring the conditions under which reinforcement might occur, Servedio and Kirkpatrick (1997) demonstrated that it is more difficult for reinforcement to operate under one‐directional as opposed to two‐directional gene flow. Therefore, it should be more common in nature to observe bidirectional hybridization and introgression.

Theory also predicts that upon formation of a reinforcement contact zone, introgression should occur at a relatively high rate initially but should decline as prezygotic isolation evolves (Blair, 1974; Britch, Cain, M. L., & Howard, D. J. 2001; Dobzhansky, 1940; Jones, 1973). Longitudinal studies of reinforcement contact zones through time are consistent with this prediction (Pfennig, 2003; Pfennig & Simovich, 2002). An additional approach for testing this prediction is to compare levels of hybridization in older versus more recent contact zones. The expected pattern is that in older contact zones, where reinforcement has had time to generate high levels of prezygotic isolation, hybridization should be rare, whereas in more recent contact zones, where prezygotic isolation is low, hybridization should be more prevalent.

Recent work has suggested that interactions between species in contact zones can not only lead to increased isolation between the two focal taxa, but via a process termed cascade reinforcement, these interactions between species can promote diversification within each of the interacting species (Howard, 1993; Ortiz‐Barrientos, Grealy, A., & Nosil, P. 2009). Intraspecific differentiation can occur due to divergent natural and sexual selection pressures across allopatric and sympatric populations (Comeault & Matute, 2016; Hoskin & Higgie, 2010; McPeek & Gavrilets, 2006; Pfennig, 2016; Pfennig & Pfennig, 2009; Pfennig & Ryan, 2006, 2007; Thompson, 2005). As a consequence reproductive behaviors may diversify across the distributions of taxa (Bewick & Dyer, 2014; Dyer, White, Sztepanacz, Bewick, & Rundle, 2013; Hoskin et al., 2005; Humphreys, Rundle, & Dyer, 2016; Kozak et al., 2015; Porretta & Urbanelli, 2012; Rice & Pfennig, 2010). Thus, we might predict that species experiencing reinforcement would also exhibit elevated levels of genetic differentiation across their geographic distributions, such as between allopatry and sympatry (Pfennig & Rice, 2014; Rice, McQuillan, Seears, & Warren, 2016). Furthermore, in cases of more complex species interactions, such as where three or more species interact across a contact zone, the divergent selection pressures may further accelerate genetic diversification across sympatric populations originating from different communities.

We tested the theoretical predictions outlined above in the North American chorus frogs (Hylidae: *Pseudacris*). Specifically, we focused on examining the contact zone between *P. feriarum* and *P. nigrita*, two species that are sympatric along the Fall Line, which separates the Coastal Plain and Piedmont regions of the southeastern United States. Phylogeographic data suggest that *P. feriarum* and P*. nigrita* diverged approximately ~8 mya (Lemmon, Lemmon, & Cannatella, 2007; Lemmon, Lemmon, Collins, Lee‐Yaw, & Cannatella, 2007; Moriarty & Cannatella, 2004) and have presumably since come into secondary contact. Statistical tests of the directionality of geographic expansion using a spatially explicit phylogeographic framework indicate that *P. feriarum* has expanded its range northward recently enough that the footprint of expansion is still present (Lemmon & Lemmon, 2008), presumably since the last glacial maximum ~10,000 years ago (Williams, Shuman, Webb, Bartlein, & Leduc, 2004; Williams, Webb, Richard, & Newby, 2000). Thus, although we cannot pinpoint the precise timing of contact, there is evidence that the southern populations of *P. feriarum* and *P. nigrita* represent older contact zones, whereas northern populations are more recent contact zones (Lemmon & Lemmon, 2008).

Geographic contact between species in this system has led to evolution of RCD in male acoustic signals and female preferences for these signals as a consequence of reinforcement. Both natural and sexual selection disfavors hybrids: Male F1 hybrids are partially sterile, and reproductive signals of male hybrids are strongly rejected by pure species females (Lemmon & Lemmon, 2010). Although sympatric populations vary geographically in both the signal trait and the species that has diverged, RCD of male acoustic signals has occurred in all sympatric populations studied to date (Lemmon, 2009). The magnitude of divergence varies substantially between the southern and northern areas of the contact zone. In the south, RCD is high and has occurred only in *P. feriarum*. In the north, the degree of RCD is low and is present only in *P. nigrita*. Studies of female mating preference behavior in *P. feriarum* from the Florida southern region indicate that female preferences have also diverged in sympatry. Putative hybrids between *P. feriarum* and *P. nigrita* with acoustically intermediate signals and intermediate phenotypes have been collected in the field in both northern and southern regions (Lemmon, 2009), but laboratory‐raised and wild‐caught F1 hybrid males are strongly rejected in female choice experiments by wild *P. feriarum* females (Lemmon & Lemmon, 2010). Although these data suggest that natural hybridization probably occurs between these species, genetic evidence has not yet been presented.

In this study, we address the following questions regarding hybridization in a reinforcement contact zone: (1) Does natural hybridization occur between *P. feriarum* and *P. nigrita* in sympatric regions? (2) Is the level of admixture higher in more recent contact zones (northern regions) compared to older contact zones (southern regions)? (3) Is gene flow bidirectional or are females of the species exhibiting stronger RCD less likely to hybridize, leading to asymmetric hybridization? (4) Do the interacting species show evidence for greater genetic differentiation between sympatric–sympatric and sympatric–allopatric localities compared to allopatric–allopatric localities? (5) Do reproductive (acoustic) behaviors predict hybrid index? (6) Does the degree of RCD predict admixture levels in populations? We address these questions utilizing nuclear, mitochondrial, and behavioral data from >1,000 individuals across >50 localities and ten focal sympatric regions across the southeastern United States. This work satisfies the final of five criteria put forth by Howard (1993) that we have tested in this system (Lemmon, 2009; Lemmon & Lemmon, 2010; Malone, Ribado, & Lemmon, 2014) to demonstrate that RCD in chorus frogs is due to reinforcement: hybridization occurs in nature.

## 2 METHODS

### 2.1 Sampling and DNA extraction

**Figure 1:**
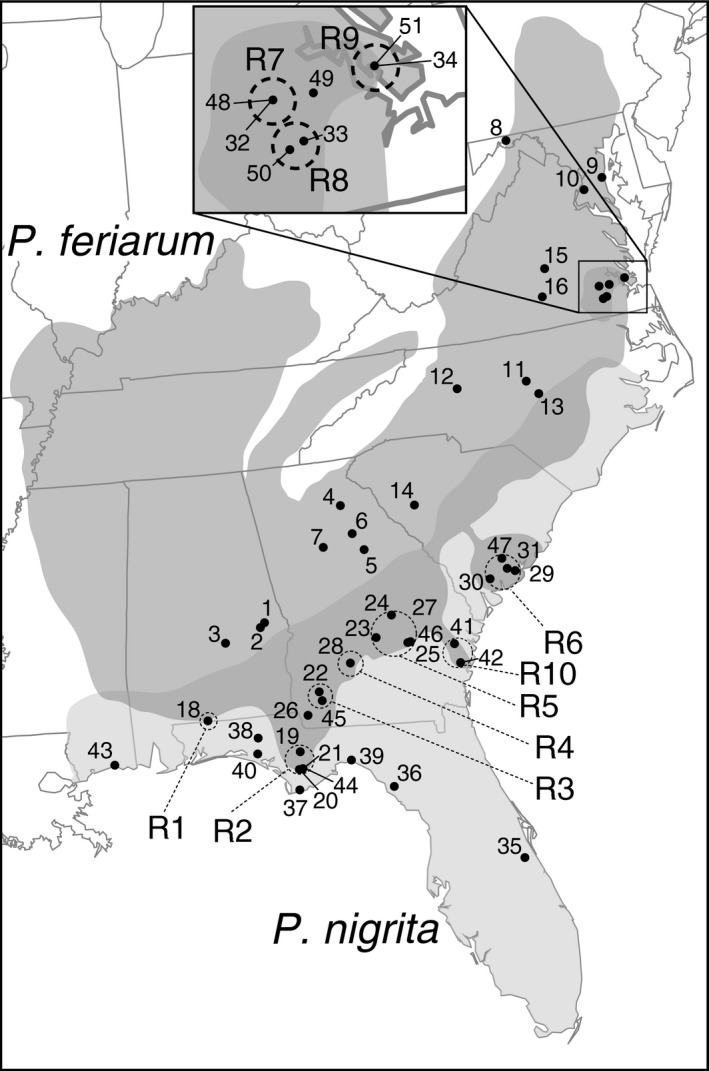
Distribution of 51 localities sampled from sympatry and allopatry across the range of *P. feriarum* and *P. nigrita*. Ten focal regions spread across the contact zone are indicated by broken lines (e.g., R1, etc.). Medium gray indicates the distribution of *P. feriarum*, light gray *P. nigrita*, and dark gray the contact zone between species. Figure modified from Lemmon (2009)

For the genetic datasets, we sampled 1,118 adult chorus frogs (*P. feriarum* and *P. nigrita*) from 51 localities (counties) across the southeastern United States (Figure 1, Table 1; Table S1). We focused here on estimating adult hybrid frequencies rather than mating frequencies. Sampling was concentrated in ten focal regions of sympatry between *P. feriarum* and *P. nigrita* (R1–R10 in Table 1). Allopatric and sympatric localities with smaller sample sizes were also included (Table S2). Note that we were unable to locate sympatric locations in northeastern South Carolina and eastern North Carolina for this study (Figure 1). Despite the presence of museum records in these areas, our extensive surveys of most of the documented historical localities failed to identify extant sympatric sites. Scientific collecting permits were obtained from all relevant states and parks. Frogs were either toe‐clipped and released or dissected for liver, leg muscle, and heart tissue. Tissues were either frozen in liquid nitrogen or preserved in tissue buffer or 95% ethanol and stored at −80°C. Specimens were deposited into the Texas Natural History Collection or the University of Florida Museum of Natural History. Genomic DNA was extracted from tissue samples using an OMEGA bio‐tek e.Z.N.A. Tissue DNA kit or a QIAGEN^®^ DNeasy Blood and Tissue kit and subsamples were diluted to 10–50 ng/μl.

**Table 1:** Localities and sample sizes of populations and ten sympatric focal regions examined

Population	Region	*N*	Locality (State, County)	Geographic group
Allopatric *P. feriarum*				
Pop001	–	18	Alabama (Lee)	Inland clade
Pop002	–	83	Alabama (Macon)	Inland clade
Pop003	–	9	Alabama (Montgomery)	Inland clade
Pop004	–	5	Georgia (Banks)	Border between clades
Pop005	–	12	Georgia (Greene)	Inland clade
Pop006	–	3	Georgia (Oglethorpe)	Border between clades
Pop007	–	2	Georgia (DeKalb)	Border between clades
Pop008	–	3	Maryland (Allegany)	Coastal clade
Pop009	–	6	Maryland (Ann Arundel)	Coastal clade
Pop010	–	2	Maryland (Harford)	Coastal clade
Pop011	–	5	North Carolina (Chatham)	Coastal clade
Pop012	–	16	North Carolina (Davie)	Coastal clade
Pop013	–	6	North Carolina (Wake)	Coastal clade
Pop014	–	10	South Carolina (Greenwood)	Coastal clade
Pop015	–	4	Virginia (Cumberland)	Coastal clade
Pop016	–	4	Virginia (Prince Edward)	Coastal clade
Sympatric *P. feriarum*				
Pop017*	R2	13	Florida (Liberty)	Lab hybrids
Pop018	R1	10	Alabama (Escambia)	Inland clade
Pop019	R2	5	Florida (Calhoun)	Inland clade
Pop020	R2	13	Florida (Gulf)	Inland clade
Pop021	R2	225	Florida (Liberty)	Inland clade
Pop022	R3	15	Georgia (Baker)	Inland clade
Pop023	R5	5	Georgia (Dodge)	Border between clades
Pop024	R5	22	Georgia (Laurens)	Border between clades
Pop025	R5	10	Georgia (Montgomery)	Border between clades
Pop026	−	2	Georgia (Seminole)	Border between clades
Pop027	R5	5	Georgia (Wheeler)	Border between clades
Pop028	R4	7	Georgia (Worth)	Border between clades
Pop029	R6	2	South Carolina (Charleston)	Coastal clade
Pop030	R6	35	South Carolina (Colleton)	Coastal clade
Pop031	R6	41	South Carolina (Dorchester)	Coastal clade
Pop032	R7	7	Virginia (Prince George)	Coastal clade
Pop033	R8	37	Virginia (Sussex)	Coastal clade
Pop034	R9	86	Virginia (York)	Coastal clade
Allopatric *P. nigrita*				
Pop035	–	5	Florida (Brevard)	Southern range
Pop036	–	2	Florida (Dixie)	Southern range
Pop037	–	6	Florida (Franklin)	Southern range
Pop038	–	6	Florida (Holmes)	Southern range
Pop039	–	12	Florida (Jefferson)	Southern range
Pop040	–	36	Florida (Walton)	Southern range
Pop043	–	13	Mississippi (Harrison)	Southern range
Sympatric *P. nigrita*				
Pop041	R10	3	Georgia (Liberty)	Southern range
Pop042	R10	23	Georgia (McIntosh)	Southern range
Pop044	R2	143	Florida (Liberty)	Southern range
Pop045	R3	9	Georgia (Baker)	Southern range
Pop046	R5	3	Georgia (Montgomery)	Southern range
Pop047	R6	9	South Carolina (Dorchester)	Southern range
Pop048	R7	22	Virginia (Prince George)	Northern range
Pop049	−	3	Virginia (Surrey)	Northern range
Pop050	R8	62	Virginia (Sussex)	Northern range
Pop051	R9	33	Virginia (York)	Northern range

Mitochondrial clades of *P. feriarum* from Lemmon, Lemmon, A. R., & Cannatella, D. C. (2007) are indicated (Inland and Coastal clades) and populations from the main *P. nigrita* (Southern) and disjunct (Northern) range are shown. An “*” indicates laboratory‐created hybrids from the region of sympatry.

### 2.2 Microsatellite genotyping and scoring

A total of 1,118 individuals were genotyped at 12 polymorphic tetra‐ and dinucleotide microsatellite loci, including some previously published markers (Lemmon, Murphy, & Juenger, 2011; Tables S3 and S4). Multiplexed PCRs (10 μl total volume) contained 3 μl nuclease‐free H_2_0, 1 μl 10× primer mix, and 5 μl QIAGEN^®^ Multiplex PCR mix, and 1 μl of diluted genomic DNA. To make 10x primer mixes for the different multiplexes, primers were combined and diluted with TE buffer to a stock concentration of 100 μm (containing each primer at 2 μm); the multiplexes are listed in Tables S3 and S4, and all forward primers were fluorescently labeled. The PCR protocol consisted of an activation step at 95°C for 15 min, followed by 35 cycles of denaturation at 94°C for 30 s, annealing at 48–56°C (depending upon the multiplex; Tables S3 and S4) for 1 min 30 s, and extension at 72°C for 1 min 30 s, and finally a final extension at 60°C for 30 min. PCR amplification products were then diluted 1:10 with nuclease‐free water. Diluted PCR product (1 μl) was combined with 10.65 μL Hi‐Di^™^ Formamide (ABI) and 0.35 μl GeneScan^™^ 500 ROX^™^ or 500 LIZ^™^ dye size standard (depending upon the multiplex) for fragment analysis on an ABI 3730 Genetic Analyzer at Florida State University. Fragment sizes were visualized as histogram distributions in R (R Project for Statistical Computing), and boundaries between peaks representing bin ranges were recorded and applied to the raw data to determine alleles (fragment lengths).

### 2.3 Microsatellite diversity analysis

We examined characteristics of the microsatellite markers for each species in all populations with *n* = 20 or larger (Table S2). Samples were pooled by species and county for all analyzes, with four exceptions. These were cases where a single individual was obtained from a county and thus was pooled with the sample from a neighboring county (ECM0180 pooled with Harford Co., MD; ECM5125 pooled with *P. feriarum* from Dorchester Co., SC; ECM5100 and ECM5095 pooled with *P. nigrita* from Dorchester Co., SC). The 51 groups are referred to as populations for analyzes below. Detailed analyzes were conducted for each microsatellite locus in the two largest reference allopatric populations: one of *P. feriarum* from Macon Co., Alabama (*n* = 83) and one of *P. nigrita* from Walton Co., Florida (*n* = 36; Tables S3, S4).

We tested the assumption of linkage equilibrium (LD) across loci using GENEPOP version 4.2 (Raymond & Rousset, 1995; Rousset, 2008; 1,000 dememorizations and one million steps of the Markov chain, 1,000 batches with 1,000 iterations per batch). We tested the assumptions of Hardy–Weinberg equilibrium (HWE) using GenoDive version 2.0b25 (Meirmans & Van Tienderen, 2004) using the heterozygosity‐based G_is_ statistic (Nei, 1987). Expected and observed heterozygosities as well as inbreeding coefficients were also calculated in GenoDive (Tables S2–S4). We utilized Micro‐Checker version 2.2.3 (Van Oosterhout, Hutchinson, Wills, & Shipley, 2004) to assess genotyping errors, such as allelic dropouts, stuttering, or null alleles.

### 2.4 Admixture analysis and comparison of hybridization levels across populations

Hybridization frequencies were estimated for all 1,118 individuals across populations using the basic admixture model in STRUCTURE (Pritchard, Stephens, & Donnelly, 2000) with the following settings: no linkage, correlated allele frequencies, burn‐in length 50,000, and 150,000 steps after burn‐in; default settings were employed for other parameters. Analyzes were run from *K* = 1 to *K* = 10 with 10 replicates of each value of assumed clusters. The optimal *K* value was determined using the method of Evanno, Regnaut, and Goudet (2005), implemented in Clumpak (Kopelman, Mayzel, Jakobsson, Rosenberg, & Mayrose, 2015). STRUCTURE plots were visualized using the Destruct for many K's feature in Clumpak.

A hybrid index was estimated for each of the five focal sympatric regions with large sample sizes (R2; R5–R6; R8–R9), as well as for five sympatric regions of *n* < 30 (R1 *n* = 10; R3 *n* = 24; R4 *n* = 7; R7 *n* = 29; R10 *n* = 26; Table 1) using the maximum likelihood‐based method GenoDive developed by Buerkle (2005). Briefly, this method utilizes the allele frequency distributions of two parental species (a reference population and an alternative population) and the genotype of the putative hybrid to estimate the hybrid index. To set a reference and alternative population, all allopatric *P. feriarum* samples were pooled into one reference group (*n* = 188), and all allopatric *P. nigrita* were pooled into a second reference group (*n* = 80). A hybrid index was then estimated for each of the ten sympatric focal regions with these references in separate analyzes.

Individuals were classified as either a hybrid or a parental species using two methods. In the first method, laboratory‐created F1 hybrids from Lemmon and Lemmon (2010; parental *P. feriarum* females × *P. nigrita* males from Liberty Co., FL, USA) were genotyped for the same microsatellite loci used in this study, and their hybrid index was estimated using the same methods and reference populations as above. The boundaries of F1 hybrid versus pure genotypes were then set based on the range of hybrid index values exhibited by these control samples (hybrid index of laboratory hybrids ranged from 0.5 to 0.75; therefore, the boundaries were set at 0.25–0.75). Thus, all wild‐sampled individuals with hybrid indices falling within the range of the laboratory hybrid controls were classified as putative F1 hybrids, although we are aware that this hybrid index range may also have included some backcross and introgressed progeny as well. Precision in estimation of F1 hybrid index is expected to improve with the inclusion of additional markers (Buerkle, 2005). In the second method, individuals were classified as hybrids of undetermined class (including but not limited to F1 hybrids) if their 95% confidence intervals estimated in GenoDive using the Buerkle (2005) approach did not extend to 0 or 1, where 0 represents the index of the first parental species, and 1 represents the index of the second parental species (following Als et al., 2011). Hybridization frequency was also estimated using NewHybrids (Anderson and Thompson 2002) under default settings. Although this program additionally provides estimates of hybrid class, we do not present these results due to insufficient power of our data to provide robust estimates as a consequence of low marker sample size.

To determine whether the frequencies of hybridization differ across the five large focal regions, we conducted a series of pairwise randomization tests. In these analyzes, we compared the proportion of individuals classified as (1) F1 hybrids and (2) any type of hybrid, using the two methods above, across the five regions. Tests were performed in the R statistical environment version 3.1.0 (R Core Team 2014). Test statistics were calculated as the difference in proportion of hybrids between pairs of populations and compared against null distributions generated from 100,000 randomizations. For each replicate from the null distribution, individuals were randomized between the pair of focal regions without replacement. A total of 10 pairwise tests were conducted using each hybrid classification method, and a sequential Bonferroni correction was performed to correct for multiple (10) tests (Rice, 1989).

Although exact dating of hybrid zone formation is beyond the scope of this study, relative timing of contact between species across regions was derived from phylogeographic data, which support recent expansion of *P. feriarum* northward into Virginia and surrounding areas and suggests relatively younger contacts in Regions 7–9 (R7–R9; Lemmon & Lemmon, 2008). This interpretation is based upon multiple statistical analyzes of *P. feriarum* mitochondrial data using a spatially explicit random‐walk model of migration across a landscape (Lemmon & Lemmon, 2008). Moreover, the ages of all contact regions examined are a minimum of 100 years old, based on morphological examination of early records of both species in museum collections (Lemmon, Lemmon, Collins, & Cannatella, 2008). In terms of the age of RCD in different populations, acoustic data obtained in the 1960s and 1970s for both species (Fouquette, 1975) indicate that RCD of male acoustic signals to current levels occurred a minimum of 50 years ago (H. Milthorpe and E. M. Lemmon, unpub. data).

### 2.5 Ascertaining the direction of successful hybridization in F1 hybrids

For individuals identified as F1 hybrids using the microsatellite‐based method above (for either the 12‐locus or 10‐locus datasets), the maternal parent was characterized through Sanger sequencing of a fragment of the 16S rRNA gene of the maternally inherited mitochondrion. The methods employed follow Moriarty and Cannatella (2004), Lemmon, Lemmon, & Cannatella, (2007) and Lemmon, Lemmon, Collins, Lee‐Yaw, J. A., & Cannatella, D. C. (2007). Briefly, partial sequence of the 16S gene (~700 bp) was obtained through amplification via polymerase chain reaction using the 16sc/16sd primers (Moriarty & Cannatella, 2004). Sequencing was performed with the 16sc primer using the ABI Big Dye terminator ready‐mix on an ABI 3730 Genetic Analyzer (Applied Biosystems). Sequences were aligned using MAFFT 7.127b (Katoh, Misawa, Kuma, & Miyata, 2002; Katoh & Standley, 2013) to the large number of previously published sequences for the two species for this gene region (Lemmon, Lemmon, & Cannatella, 2007; Lemmon, Lemmon, Collins, Lee‐Yaw, J. A., & Cannatella, D. C. 2007; Moriarty & Cannatella, 2004), and a genus‐wide phylogeny was generated using RAxML‐III version 8.0.0 (Stamatakis, Ludwig, & Meier, 2005; GTRCAT model, 1,000 bootstrap replicates, *Hyla chrysoscelis* as outgroup) with up to five published reference sequences per species to establish the species of origin for the mitochondrial genome in each F1 hybrid. Of the 190 F1 hybrids identified using microsatellites, sufficient DNA remained to sequence 185 for the 16sc mitochondrial regions. Five additional putative F1 hybrids (based on morphology and acoustic data) from R6 (*n* = 1) and R8 (*n* = 4) were also sequenced, though not genotyped. To determine whether there was evidence for asymmetric introgression (i.e., whether the two possible maternal parents occur in unequal frequencies), exact binomial tests were performed on localities with the number of F1 hybrids >15 individuals: (1) Florida R2 individuals (*n* = 109), (2) Virginia R8 (*n* = 26), (3) Virginia R9 (*n* = 16), (4) Georgia R10 (*n* = 18), and (2) all regions combined (*n* = 190).

### 2.6 Genetic differentiation within species

To further examine genetic differentiation within species, principal coordinates analyses (PCoAs) were performed on microsatellite data (binned fragment lengths) from: (1) both species together, (2) *P. feriarum* only, and (3) *P. nigrita* only. Analyzes were conducted in GenAlEx 6.5 (Peakall & Smouse, 2006, 2012) on a genetic distance matrix (*R*_st_; Slatkin, 1995) using the covariance‐standardized PCoA method. Scores from the first three PCoA axes were saved, and graphs were plotted in R.

The degree of isolation‐by‐distance (IBD; correlation between genetic and geographic distance) was tested using a Mantel test (Mantel, 1967; Smouse, Long, & Sokal, 1986; with 10,000 permutations) in Arlequin version 3.5 (Excoffier & Lischer, 2010). The test was performed using *F*_st_ values (Wright, 1951, 1965, 1978) between populations with *n* ≥ 5 calculated in Arlequin and with Euclidean geographic distances between populations calculated in Geographic Distance Matrix Generator v1.2.3 (Ersts, 2013) using GPS coordinates. Prior to analysis, all hybrids identified using both hybrid index methods described above were removed from sympatric populations. IBD analyzes were performed separately on the two species. In order to test for significantly lower IBD among allopatric population pairs, a randomization test was performed in which the residual *F*_st_ values from the IBD analysis were computed and the test statistic was calculated as the difference between the average residual of comparisons involving sympatric populations (sympatric–sympatric or sympatric–allopatric) and the average residual of comparisons involving only allopatric populations (allopatric–allopatric). The null distribution was estimated by recomputing the test statistic after randomizing the assignment of allopatry or sympatry to each locality. A total of 200 randomizations were performed, and the test statistic was compared to the null distribution.

### 2.7 Using acoustic signal information to predict hybrid index and admixture levels

Acoustic signal data were taken from the Lemmon (2009) dataset (*n* = 318) and from additional frogs recorded (*n* = 155) since publication of the study (*n* = 473 total; Table S1). A total of 185 sympatric individuals for which acoustic data were available were genotyped for the microsatellite loci described above. These individuals included the laboratory‐created F1 hybrids from R2 and wild‐caught frogs from nine of the 10 focal regions in this study, spread across the zone of sympatry between *P. feriarum* and *P. nigrita*. New acoustic mating signals (advertisement calls) were collected and analyzed following Lemmon (2009). Population‐specific temperature corrections were applied to the expanded dataset following Lemmon (2009) by performing linear regression of temperature vs. the call variable to estimate the slope. This information was then used to correct the call characters influenced by temperature to a common temperature of 14°C across all individuals in the population prior to data analysis.

To determine the degree that acoustic characteristics predict hybrid index, linear regression analyzes were performed on two datasets. In these analyzes, pulse rate and pulse number, the two acoustic characters that show reproductive character displacement in sympatry (Fouquette, 1975; Lemmon, 2009) were each regressed against hybrid index. The first dataset consisted of 75 individuals from R2‐R3 (Liberty and Gulf Cos., FL and Baker Co. GA). These regions were combined because of their geographic proximity and large sample sizes; other regions were not examined separately due to the paucity of available hybrids with matching calls in these areas. The second dataset included all 185 individuals (above) combined across all the regions (except R7, where data were not available). Acoustic characters were log‐transformed prior to analysis. A stepwise multiple linear regression analysis was also performed with pulse rate and pulse number to determine: (1) which of the two acoustic variables is a better predictor of hybrid index and (2) whether both acoustic variables together significantly improve prediction of hybrid index. All statistical analyzes were performed in JMP version 10.0.0 (SAS Institute Inc., 2012).

To assess the relationship between degree of RCD and admixture, linear regressions were performed on *P. feriarum* and *P. nigrita* separately, using sympatric regions as the unit of replication (*n* = 8 and *n* = 6, respectively). Because these analyzes did not require individual genotypes to be matched to acoustic signals, a broader dataset from 441 published and unpublished acoustic recordings was utilized (Table S1), primarily taken from Lemmon (2009). Admixture levels for each region were calculated via the F1 hybrid method and the CI method described above. In addition an “advanced hybrids” admixture metric was calculated by subtracting the number of F1s from the number of undetermined hybrids and calculating the proportion of advanced‐generation hybrids (CI‐F1 method). Acoustic distances were quantified by averaging pulse rate and pulse number for all allopatric individuals combined and for each region separately, by species, and calculating the Euclidean distance of the two acoustic variables between each sympatric region and allopatry, where the allopatric character state was considered to be the baseline nondisplaced signal. Regressions of degree of RCD versus admixture were carried out separately for *P. feriarum* and *P. nigrita*. All analyzes were conducted in JMP.

## 3 RESULTS

### 3.1 Genetic variation in microsatellite loci

All 12 of the microsatellite loci had high levels of variation, ranging from 6 to 32 alleles per locus (mean = 20) in the allopatric *P. feriarum* population from Macon Co., AL and from 6 to 21 alleles per locus (mean = 13) in the allopatric *P. nigrita* from Walton Co., FL (Tables S3 and S4). A total of 592 alleles were found across all loci in our sample of 1,118 individuals. Deviations from Hardy–Weinberg Equilibrium were not detected in any of the populations with *n* = 20 or more individuals after a table‐wide sequential Bonferroni correction for multiple tests (Rice, 1989). Linkage disequilibrium between pairs of loci was not detected in either of the allopatric reference populations after a Bonferroni correction but was identified in 1–2 pairs of loci in four of the sympatric populations (Table S2). Evidence for null alleles was detected in 6 and 8 loci in the reference allopatric *P. feriarum* and *P. nigrita* populations, respectively. The frequency of nulls, however, varied across species and loci. In *P. feriarum*, null frequency was ≤10% in 10 of 12 loci, whereas in *P. nigrita*, null frequency was ≤10% in 6 loci and ≤15% in 10 of 12 loci. The two loci with 30% or higher null frequency in *P. nigrita* were the same loci with >10% nulls in *P. feriarum* also (13% at P_fer_c101070 and 21% at P_fer_lrc46999 in *P. feriarum*). Therefore, analyzes were conducted with and without these two loci to ascertain their effects on the results. As the effect was minimal, most results are presented based on the 12‐locus dataset only.

### 3.2 Comparison of hybridization levels across populations

**Figure 2:**
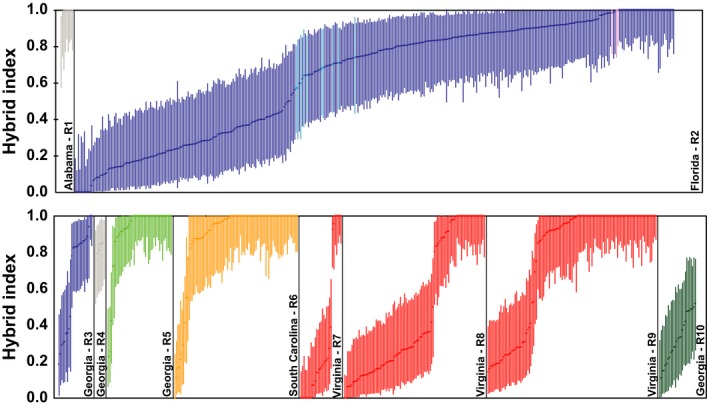
Variation in hybrid indices within and across ten sympatric regions, showing hybrid index (points) and 95% confidence intervals on estimates. A hybrid index of “0” indicates a pure *P. nigrita*, a “1” indicates pure *P. feriarum*, and intermediate values indicate potential hybrids. In Florida R2, turquoise indicates laboratory‐created F1 hybrids and pink indicates laboratory‐created pure *P. feriarum*, whereas dark blue indicates wild‐caught individuals

We detected evidence of natural hybridization in all ten sympatric focal regions of *P. feriarum* and *P. nigrita* sampled along their contact zone throughout the southeastern U.S. in Alabama, Florida, Georgia, South Carolina, and Virginia. STRUCTURE analyzes identified the presence of two main clusters in the dataset, corresponding to *P. feriarum* and *P. nigrita* (Fig. S1); *K* = 2 was the best‐supported model based on the Evanno, Regnaut, S., & Goudet, J. (2005) Δ*K* method (Fig. S2). GenoDive analyzes identified genotypes consistent with F1 hybrids in 9 of 10 regions and hybrids of undetermined class in all regions (Figure 2; Table S1). The proportion of F1 hybrids ranged from 5% in South Carolina populations to 31% in Florida (Table 2). The proportion of hybrids of undetermined class (which includes F1 hybrids also) varied from 11% in South Carolina populations to 78% in Florida (Table 3). NewHybrids analyzes detected evidence for hybrids of different classes in 7 of 10 regions (Table S5) and in all regions with a sample size >30. Under a 95% posterior probability threshold, the proportion of hybrids of varied from ~1% in South Carolina and Virginia to 5.3% in Florida (Table S5).

**Table 2:** Comparison of proportion of F1 hybrids across populations via pairwise randomization tests

Sympatric Pop. 1	Prop. Hyb 12 loci	Prop. Hyb 10 loci	*N*	Sympatric Pop. 2	Prop. Hyb 12 loci	Prop. Hyb 10 loci	*N*	*p* value 12 loci	*p* value 10 loci
Florida R2	0.31	0.35	386	South Carolina R6	0.05	0.02	87	<.00001*	<.00001*
Florida R2	0.31	0.35	386	Georgia R5	0.09	0.07	45	.00076*	.00004*
Florida R2	0.31	0.35	386	Virginia R8	0.23	0.20	99	.07818	.00290*
Florida R2	0.31	0.35	386	Virginia R9	0.15	0.03	119	.00029*	<.00001*
South Carolina R6	0.05	0.02	87	Georgia R5	0.09	0.07	45	1	1
South Carolina R6	0.05	0.02	87	Virginia R8	0.23	0.20	99	.00035*	.00006*
South Carolina R6	0.05	0.02	87	Virginia R9	0.15	0.03	119	.01493	.69230
Georgia R5	0.09	0.07	45	Virginia R8	0.23	0.20	99	.03024	.03360
Georgia R5	0.09	0.07	45	Virginia R9	0.15	0.03	119	.21913	1
Virginia R8	0.23	0.20	99	Virginia R9	0.15	0.03	119	.57349	.00194*

Results are shown from 12‐loci and 10‐loci analyzes. Populations with significantly different hybridization frequencies after a sequential Bonferroni correction are indicated by an “*”.

**Table 3:** Comparison of proportion of hybrids of undetermined class across populations via pairwise randomization tests

Sympatric Pop. 1	Prop. Hyb 12 loci	Prop. Hyb 10 loci	*N*	Sympatric Pop. 2	Prop. Hyb 12 loci	Prop. Hyb 10 loci	*N*	*p* value 12 loci	*p* value 10 loci
Florida R2	0.78	0.76	386	South Carolina R6	0.11	0.09	87	<.00001*	<.00001*
Florida R2	0.78	0.76	386	Georgia R5	0.31	0.24	45	<.00001*	<.00001*
Florida R2	0.78	0.76	386	Virginia R8	0.67	0.60	99	.01572	.00127*
Florida R2	0.78	0.76	386	Virginia R9	0.36	0.33	119	<.00001*	<.00001*
South Carolina R6	0.11	0.09	87	Georgia R5	0.31	0.24	45	.87045	.8735
South Carolina R6	0.11	0.09	87	Virginia R8	0.67	0.60	99	<.00001*	<.00001*
South Carolina R6	0.11	0.09	87	Virginia R9	0.36	0.33	119	.00002*	.00002*
Georgia R5	0.31	0.24	45	Virginia R8	0.67	0.60	99	.00009*	.00009*
Georgia R5	0.31	0.24	45	Virginia R9	0.36	0.33	119	.34276	.19974
Virginia R8	0.67	0.60	99	Virginia R9	0.36	0.33	119	.05069	.08635

Results are shown from 12‐loci and 10‐loci analyzes. Populations with significantly different hybridization frequencies after a sequential Bonferroni correction are indicated by an “*”.

Comparison of hybridization frequencies across all 10 pairs of focal populations with *n* > 30 indicated that hybridization rates vary substantially among populations. A significantly higher proportion of F1 hybrids was present in Florida R2 than in the other focal populations, except Virginia R8, based on the method of classifying putative F1 hybrids using laboratory cross‐data (Table 2). Further, more F1 hybrids were identified in Virginia R8 than South Carolina R6. A significantly higher proportion of hybrids of any class were also detected in Florida R2 compared to the other focal populations, except Virginia R8, based on the CI method of classifying hybrids (Table 3). Additionally, a higher frequency of hybridization was identified in Virginia R8 and R9 compared to both South Carolina R6 and Georgia R5, although the latter difference was not significant for Virginia R9.

Results from the 12 versus 10 loci analyzes were essentially the same, with similar hybrid proportions estimated from both datasets. The primary discrepancies were a significant difference in hybrid proportion between Florida R2 and Virginia R8 and between Virginia R8 and Virginia R9 detected in the 10 loci but not the 12 loci analysis of F1s (Table 2) and a significant difference between Florida R2 and Virginia R8 detected in the 10 loci but not the 12 loci analysis of hybrids of any class (Table 3).

### 3.3 Direction of successful hybridization in F1 hybrids

Mitochondrial sequencing revealed both types of F1 hybrid crosses in natural populations (Table 4; Fig. S3; Table S1). Exact binomial tests showed evidence for asymmetric introgression, however, with *P. nigrita* serving as the maternal parent in most putative F1 crosses from regions that contained >15 F1 hybrids, including Florida R2 (*p* = 2.38e−04), Virginia R8 (*p* = 1.05e−05), Georgia R10 (*p* = 7.63e−06), but this pattern was not significant in Virginia R9 (*p* = 8.04e−01). Overall, when all regions were combined there was strong evidence that *P. nigrita* is the primary maternal parent in F1 hybrids (*p* = 1.85e−11). Small sample sizes for F1s in six localities precluded an in depth examination of geographic variation in direction of introgression across regions.

**Table 4:** Direction of hybridization in F1s ascertained from mitochondrial sequencing

Region	*N*	*P. feriarum*	*P. nigrita*	*p* value
Alabama R1	1	1	0	–
Florida R2	109	35	74	2.38e−04*
Georgia R3	7	0	7	–
Georgia R4	–	–	–	–
Georgia R5	4	2	2	–
South Carolina R6	4	1	3	–
Virginia R7	2	0	2	–
Virginia R8	26	2	24	1.05e−05*
Virginia R9	16	7	9	8.04e−01
Georgia R10	18	0	18	7.63e−06*
Total	187	48	139	1.85e−11*

Number of individuals having each maternal parent is indicated and the statistical significance from exact binomial tests (“*” indicates a significant test). Results from localities with *n* > 15 are shown (“—” indicates localities with smaller sample sizes).

### 3.4 Genetic differentiation within species

**Figure 3:**
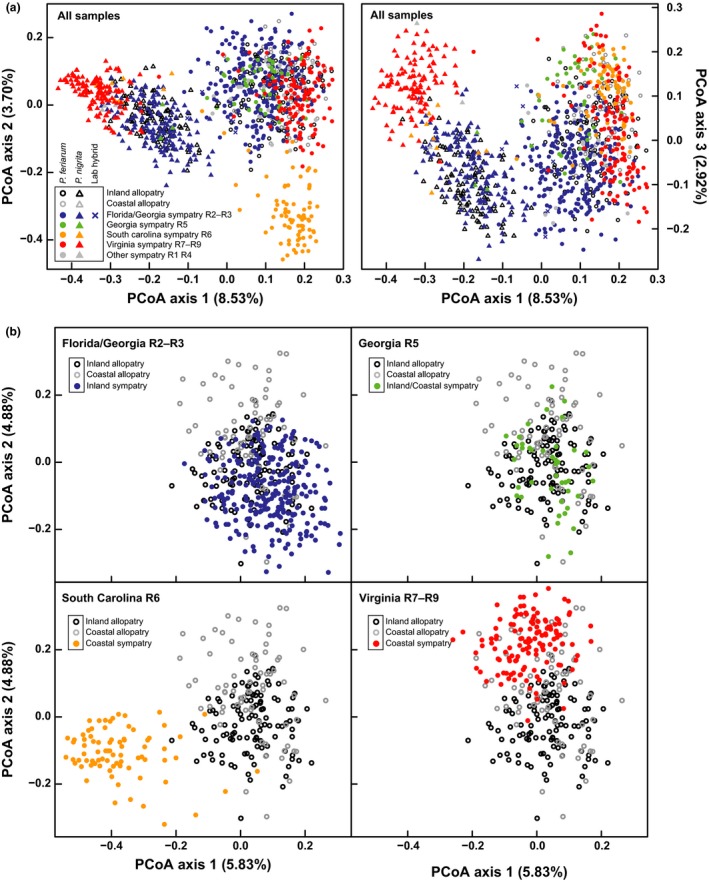
Inter‐ and intraspecific genetic variations illustrated via principal coordinates analyzes (PCoA). Sympatric and allopatric samples are indicated by solid and hollow symbols, respectively; light gray and black circles indicate Coastal Clade and Inland Clade allopatric *P. feriarum*, respectively. Colors show regions from sympatry. (a) The first PCoA analysis includes all regions and populations of *P. feriarum* (circles) and *P. nigrita* (triangles) and their hybrids (triangle or circle, based on morphological identification), with the first three axes shown. (b) The second analysis includes all regions and populations of *P. feriarum* only, with each sympatric region set shown in color relative to allopatric individuals from the two clades: R2–R3, R5, R6, and R7–R9. Note that Georgia R5 is located at the boundary between the Coastal and Inland clades and thus includes sympatric individuals from both clades. A comparable analysis for *P. nigrita* only is shown in Fig. S4

Substantial intraspecific genetic differentiation was detected within species. In the PCoA of both species together, the first axis explained 8.53% of the variation, showing nearly complete separation between species. The second axis explained 3.70% of the variation, indicating strong intraspecific differentiation within *P. feriarum* between sympatric South Carolina R6 and other *P. feriarum* (Figure 3a). The third axis explained 2.92% of the variation and showed intraspecific differentiation within *P. nigrita* between sympatric Virginia populations (Prince George R7, Sussex R8, and York R9 Counties) and all other *P. nigrita* (Figure 3a). Gene flow is likely to be reduced from the main species distributions for *P. feriarum* in South Carolina R6 and for *P. nigrita* in Virginia R7–R9 since these islands form “peninsulas” or “islands” with respect to the range of the remainder of each species.

Intraspecific differentiation is also illustrated by results of the STRUCTURE analyzes (Fig. S1). At *K* = 3 for the full dataset (both species), *P. feriarum* shows differentiation into an Inland and Coastal clade, which was previously described by Wright and Wright (1949) using morphology and by Lemmon, Lemmon, Collins, & Cannatella, D. C. (2007) based on mitochondrial markers. At *K* = 4, further substructure within the Coastal clade of *P. feriarum* consists of differentiation of South Carolina populations in deep sympatry near Charleston from the rest of the Coastal clade. This pattern is not unexpected because these populations are distinct from other *P. feriarum* with respect to their mating behaviors (Lemmon, 2009). At *K* = 5, *P. nigrita* shows differentiation between the geographically isolated populations in Virginia and the rest of the species. At *K* = 6, substructure within the Inland clade of *P. feriarum* is present between Apalachicola River floodplain populations in deep sympatry and the remainder of the Inland clade.

In the analysis of only *P. feriarum*, the first PCoA axis explained 5.83% of the variation, showing separation again of sympatric South Carolina frogs from the rest of the species (Figure 3b). The second axis, explaining 4.88% of the variation, indicated some differentiation between two mitochondrial groups previously identified within *P. feriarum*, the Coastal and Inland Clades (Lemmon, Lemmon, & Cannatella, 2007; Lemmon, Lemmon, Collins, Lee‐Yaw, J. A., & Cannatella, D. C. 2007). These two groups are parapatric or partially sympatric with respect to each other in central Georgia, approximately bounded by the Altamaha River and tributaries. The Inland group identified here includes both sympatric and allopatric populations south and west of this boundary, and the Coastal group includes both sympatric and allopatric populations north of the boundary, with the exception of the distinct sympatric South Carolina frogs from the Charleston region. In the analysis of only *P. nigrita*, the first and second axes explained 6.87% and 3.39% of the variation, respectively. The only strong population differentiation occurred along the first axis, again between Virginia and all other *P. nigrita* populations (Fig. S4).

A weak pattern of IBD was detected for *P. feriarum* using the Mantel test (Fig. S5; *r* ^2^ = .048, *p* = .0038). Strong IBD was found in *P. nigrita* (Fig. S6; *r* ^2^ = .550, *p* < .0001). The IBD randomization test indicated that in *P. feriarum*, genetic divergence between allopatric locality pairs was lower than between other types of locality pairs (sympatric–allopatric and sympatric–sympatric) after controlling for genetic distance (one‐tailed test; *p* = .054). This pattern was not observed in *P. nigrita* (*p* = .94).

### 3.5 Acoustic Signals, Hybrid Index, and Admixture Levels

**Figure 4:**
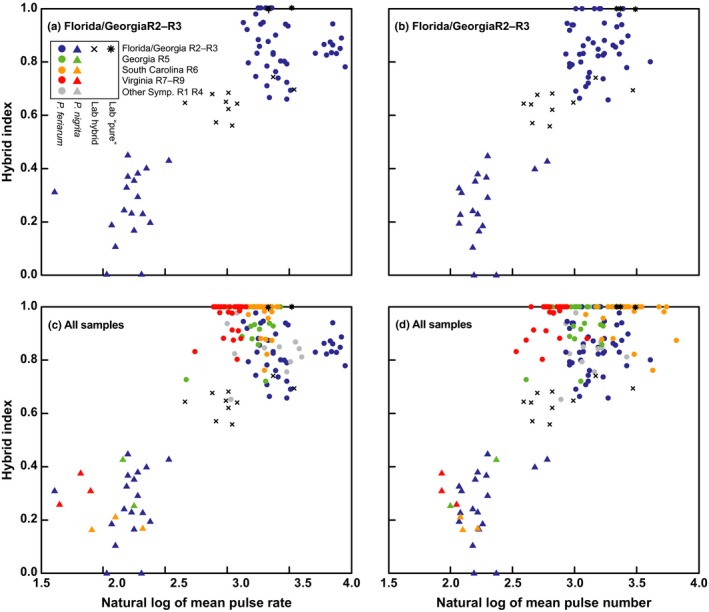
Acoustic variables predict hybrid index in sympatry. Results are shown from the 75‐individual dataset (R2–R3) for pulse rate (a) and pulse number (b) and from the 181‐individual dataset (R1–R6 and R8‐R9 combined) for pulse rate (c) and pulse number (d). Species and population symbols and colors are indicated by key and are as in Figure 3

Acoustic variables strongly predict hybrid index, individually and in combination. For both the 75‐ and 185‐individual datasets, a significant linear relationship was found between pulse rate and hybrid index (*r* ^2^ = .72, *p* < .0001 and *r* ^2^ = .57, *p* < .0001, respectively; Figure 4a,c) and between pulse number and hybrid index (*r* ^2^ = .77, *p* < .0001 and *r* ^2^ = .60, *p* < .0001, respectively; Figure 4b,d). The stepwise multiple regression indicated that pulse number is a better predictor of hybrid index than pulse rate for both datasets (Table S6). The best model, however, included both variables (*r* ^2^ = .79, *p* < .0001 and *r* ^2^ = .63, *p* < .0001, respectively) according to both the Akaike Information Criterion (AIC; Akaike, 1974) and Bayesian Information Criterion (BIC; Schwarz, 1978; Table S7).

Level of RCD was not a strong predictor of degree of admixture in sympatric regions in either *P. feriarum* or *P. nigrita*. For *P. feriarum*, RCD did not predict admixture with respect to undetermined (*r* ^2^ = .13, *p* = .38, *b* = 0.31) and advanced hybrids (*r* ^2^ = .14, *p* = .37, *b* = 0.30), or F1 hybrids (*r* ^2^ = 1.64e−3, *p* = .92, *b* = 0.01). For *P. nigrita*, RCD did not predict admixture in terms of undetermined hybrids (*r* ^2^ = .16, *p* = .43, *b* = −0.83), advanced hybrids (*r* ^2^ = .18, *p* = .40, *b* = −0.57), or F1 hybrids (*r* ^2^ = .12, *p* = .50, *b* = −0.26). Sampling of additional sympatric regions would improve the power (*P. feriarum n* = 8 and *P. nigrita n* = 6 sympatric regions) of these analyzes.

## 4 DISCUSSION

The presence of hybridization in all sympatric regions spanning the chorus frog contact zone indicates that the opportunity for reinforcement to promote the evolution of reproductive isolation exists throughout sympatry. Moreover, in all regions examined to date, premating isolation has increased in sympatry: A pattern of reproductive character displacement in male signals is present in one of the interacting species (Lemmon, 2009), which is driven by reinforcement (Lemmon & Lemmon, 2010; Malone, Ribado, J., & Lemmon, E. M. 2014). The frequency of hybridization varies considerably across the contact zone, from 5% to 31% F1 hybrids and 11% to 78% hybrids of undetermined class, suggesting a wide range of hybridization frequencies under which reinforcement may operate.

### 4.1 Evidence for geographic variation in hybridization frequencies

A striking finding from this study is the high percentage of hybrids in Florida and Virginia sympatric populations (R2 and R8; 31% and 23% F1 hybrids, respectively, and 78% and 67% undetermined class hybrids, respectively) compared to other regions (Tables 2 and 3). These estimates are also outside the range of frequencies estimated in another well‐studied frog reinforcement contact zone (i.e., 0.3%–6% F1 hybrids; Pfennig & Simovich, 2002; Pfennig, 2003). One question is whether these estimates reflect historical or present‐day hybridization, or more specifically, was there a high rate of hybridization upon initial secondary contact followed by a decline in rate through time? Although difficult to disentangle from our data, the evidence from F1 hybrids, which primarily reflects present‐day hybridization, suggests that a high level of hybridization is ongoing in these regions and is substantially higher than other regions in the contact zone (Tables 2 and 3; Figure 2). Previous work in Florida R2 indicates that F1 hybrids have high viability fitness (*s* = 0.14), but males experience strong negative sexual selection (*s* = −0.95) due to their intermediate acoustic mating signals, as well as partial sterility (*s* = −0.23; Lemmon & Lemmon, 2010). Thus, although many F1 hybrids may be produced, far fewer are able to successfully acquire a mate and produce viable offspring. Therefore, the high percentage of undetermined class hybrids is likely the result of many generations of backcrossing by the few F1 hybrids that pass through the sieve of sexual and natural selection after the first‐hybrid generation.

Results from this study do not support the prediction that putative recent contact zones have higher rates of hybridization. This prediction was derived from the expectation that hybridization rates should decline through time as reinforcement proceeds (Blair, 1974; Britch, Cain, M. L., & Howard, D. J. 2001; Coyne & Orr, 2004; Dobzhansky, 1940; Jones, 1973; Nosil, 2012; Pfennig, 2003). Instead the youngest contacts (Lemmon & Lemmon, 2008) had moderate (Virginia R8‐R9) levels of hybridization, whereas older contacts varied from high (Florida R2) to low (South Carolina R6 and Georgia R5; Tables 2 and 3; Figure 2). These data suggest: (1) relative timing of contact alone is not sufficient to explain variation in hybridization frequencies in sympatric regions, and (2) in accord with other studies (e.g., Matute, 2010; Pfennig, 2003), RCD can evolve rapidly relative to the decay of the phylogeographic footprint following range expansion. Thus, the current hybridization rates across populations likely either reflect equilibrium levels after contact rather than a spectrum of rates from early to established contact zones or else other factors have influenced hybridization frequencies across populations (Borge et al., 2005).

There are a number of additional possible explanations beyond timing of contact for variation in hybridization frequencies across the contact zone. First, hybrid incompatibilities may vary across geography, such that selection against hybridization is stronger in some regions or acts at different life history stages across areas (Parris, 2001; Sætre, Kr.l, M., Bureš, S., & Ims, R. A. 1999; Sweigart, Mason, & Willis, 2007; Veen et al., 2001). In chorus frogs, even males derived from the same population vary substantially in levels of hybrid sterility, lending support for this hypothesis (Lemmon & Lemmon, 2010). Second, ecological selection against hybridization may vary (Gow, Peichel, & Taylor, 2006; Taylor, Boughman, J. W., Groenenboom, M., Sniatynski, M., Schluter, D., & Gow, J. L. 2006). If some habitats where hybridization occurs are more favorable to survival than others, we would detect apparent differences in rates that do not reflect the actual frequency of heterospecific mating. Third, relative densities or demographic histories of the interacting species may vary geographically, thereby affecting the opportunity for heterospecific mating (Howard, 1993; Noor, 1995; Nosil, Crespi, B. J., & Sandoval, C. P. 2003; Peterson et al., 2005; Servedio & Kirkpatrick, 1997; Servedio & Noor, 2003; Yukilevich, 2012). Chorus frog contact regions vary in spatial structure from shallow sympatry, where the two species co‐occur in roughly even frequencies (Alabama R1, Georgia R3–R5) to peninsular‐type sympatric distributions where gene flow from allopatry is restricted (*P. feriarum* in Florida R2 and South Carolina R6), to island‐type sympatric distributions (*P. nigrita* in Virginia R7–R9; Figure 1). The species with the island‐ or peninsular‐distribution in a sympatric region is likely the rarer of the two in those areas, although this is extremely difficult to quantify in chorus frogs due to their transient presence in the breeding sites. Thus, the opportunity for interaction varies widely, potentially contributing to spatial variation in hybridization rates. Finally, the presence of different chorus frog species in local communities across geography may affect the rate of hybridization. For example, the presence of congener *P. brimleyi* (R6–R9; Lemmon, 2009), which has been observed mating with *P. feriarum* in nature (D. B. Means and E. Moriarty Lemmon, unpub. data), is predicted to contribute further to narrowing of the female *P. feriarum* preference function, resulting in even lower hybridization in these three‐species regions (McPeek & Gavrilets, 2006; Pfennig & Ryan, 2006, 2007). The very low‐hybridization frequency in South Carolina R6 may be related to the high density of *P. brimleyi* in this region.

### 4.2 Support for asymmetric hybridization

Evidence for asymmetric hybridization and proximal mtDNA transfer (Near et al., 2011) was detected in our data in three of the four regions with *n* > 15 putative F1 hybrids (Florida R2, Virginia R8, Virginia R9, and Georgia R10), and in the total dataset (*n* = 187; Table 4). Although asymmetric, hybridization was also bidirectional, which is consistent with the theory of Servedio and Kirkpatrick (1997), who demonstrated that reinforcement operates under a broader set of conditions when hybridization occurs in both directions. In all significant tests, the majority of mitochondrial haplotypes found in F1s belonged to *P. nigrita*, providing support that this species serves as the maternal parent in hybrid crosses more frequently than *P. feriarum*. These results are consistent with expectations, particularly in Florida R2, which is a well‐studied reinforcement contact zone (Lemmon, 2009; Lemmon & Lemmon, 2010). In Florida, female *P. feriarum* have evolved increased conspecific mating preferences in sympatry as a consequence of strong selection against hybridization with *P. nigrita* (*s* = −0.44 lifetime fitness of F1 hybrids; Lemmon & Lemmon, 2010). Thus, the observation of relatively few *P. feriarum* serving as the maternal parent in F1 crosses is consistent with these previous studies. Our data, however, cannot address whether the attempted mating rate is symmetric, even though evidence suggests that successful mating rate is asymmetric.

The concordance of asymmetric gene flow and asymmetric RCD found here is consistent with several recent studies. In a meta‐study of >600 *Drosophila* species pairs, Yukilevich (2012) found that sympatric species overwhelmingly manifested concordant isolation asymmetries: The species with stronger postzygotic isolation was also the species that experienced higher prezygotic isolation. Assuming that females are the sex that experiences a greater cost to hybridization (Lemmon & Lemmon, 2010), a natural prediction is fewer females from the species under stronger selection should engage in heterospecific mating, thus leading to asymmetric hybridization. Further, Hoskin, Higgie, M., McDonald, K. R., & Moritz, C. (2005) and Peterson et al. (2005) found a link between asymmetric gene flow and asymmetric RCD in frogs and beetles, respectively—the females of species exhibiting higher levels of RCD hybridized rarely, if ever, compared to females from the other species. Collectively, this work and the present study suggest that asymmetry in the cost of hybridization causes the species bearing the greater cost to diverge in reproductive behaviors and subsequently hybridize less due to refinement of the female preference.

### 4.3 Genetic diversification within species

Consistent with theoretical predictions (McPeek & Gavrilets, 2006; Pfennig & Ryan, 2006, 2007), we found that within *P. feriarum*, genetic divergence is higher between conspecific localities where one or both has been reinforced compared to nonreinforced localities, after accounting for geographic distance (Figure 3). This pattern is expected when cascade reinforcement between species indirectly drives diversification within species, such as between allopatric and sympatric conspecific populations. Although alternative explanations are possible, we falsified a primary alternative by ruling out the action of sensory drive in this system (Malone, Ribado, J., & Lemmon, E. M. 2014). Evidence for cascade reinforcement (Hoskin & Higgie, 2010; Howard, 1993; Ortiz‐Barrientos, Grealy, A., & Nosil, P. 2009) is accumulating rapidly across a taxonomically broad set of organisms (Bewick & Dyer, 2014; Dyer et al., 2013; Higgie & Blows, 2008; Hoskin, Higgie, M., McDonald, K. R., & Moritz, C. 2005; Humphreys, Rundle, H. D., & Dyer, K. A. 2016; Kozak et al., 2015; Pfennig & Rice, 2014; Porretta & Urbanelli, 2012; Rice & Pfennig, 2010; Rice et al., 2016; Richards‐Zawacki & Cummings, 2011). Our data are consistent with the expectation that cascade reinforcement cannot only promote the rapid divergence of reproductive behaviors among different populations (Lemmon, 2009) but also drive intraspecific genetic divergence at neutral loci (Rice & Pfennig, 2010).

Isolation‐by‐distance analyzes indicated that geographic distance explains ~55% of the genetic divergence across populations in *P. nigrita*, but only ~5% of the divergence in *P. feriarum* (Figs. S5, S6). The *P. feriarum* populations showing the greatest deviation from IBD and highest differentiation in the PCoA are from South Carolina R6, which has a sympatric “peninsula” type distribution near the Charleston, SC area (Figures 1 and 3; Schwartz, 1957). The PCoA results as well as the STRUCTURE results (Fig. S1, *K* = 4) suggest that these populations have low levels of gene flow with allopatric *P. feriarum* to the northwest along the floodplains of the Wateree and Congaree Rivers above Lake Marion. Our surveys, however, have not detected large populations along this corridor (E. Moriarty Lemmon, unpub. data). In *P. nigrita*, the populations showing the highest genetic differentiation are from a disjunct sympatric “island” relative to the main distribution of the species, R7‐R9 in eastern Virginia (Figures 1 and 3a; Fig S1; *K* = 5). These sympatric populations are ~200 mi from the current main distribution of the species, although museum records suggest that the distance was less in the last century. In both areas, RCD of the “peninsula” or “island” species rather than the widespread species has occurred, and the resulting displaced acoustic signals are not only divergent from the heterospecific taxon but also from other allopatric and sympatric conspecific populations (Lemmon, 2009). These data suggest that both behavioral divergence due to species interactions and geographic separation may be contributing to speciation in this system.

### 4.4 Acoustic signals, hybrid index, and admixture levels

The two acoustic characters that have undergone RCD in sympatry, pulse rate and pulse number, strongly predict hybrid index (Figure 4), and the continuous nature of these characters suggest they are quantitative traits. The genetic basis of acoustic signals in frogs, however, is unknown. Frog calls are complex signals, composed of multiple traits that convey different types of information to females (Gerhardt & Huber, 2002). Some components of these signals are controlled by the frog's morphology and others by its physiology or behavior (Cocroft & Ryan, 1995; Ryan, 1988). Thus, the genetic architecture underlying pulse rate and pulse number is expected to be complex, potentially involving many genes. What is known about the genomic basis of frog calls is that gene dosage affects frog signals—ploidy level is correlated with trait values (Guignard, Büchi, Gétaz, Betto‐Colliard, & Stöck, 2012; Hoffman & Reyer, 2013; Keller & Gerhardt, 2001; Mable & Bogart, 1991; Tucker & Gerhardt, 2012). More is known about the genetic architecture of acoustic signals in insects—for example, in crickets several quantitative trait loci have been identified that control pulse rate (Ellison, Wiley, & Shaw, 2011; Shaw & Lesnick, 2009; Shaw, Parsons, & Lesnick, 2007). The availability of cost‐effective genomic approaches and increasing number of assembled whole genomes are now making it more feasible to study the genomic architecture of acoustic signals in organisms with large genomes such as frogs.

The lack of relationship between level of RCD relative to allopatry and degree of admixture is potentially due to low power for both species (*P. feriarum*,* n* = 8 and *P. nigrita*,* n* = 6), but alternatively, the relationship between these variables may be subtle. For example, it is likely that the direction of behavioral phenotypic evolution matters more than absolute magnitude of RCD. In *P. feriarum*, the locality with the lowest hybridization level (South Carolina R6) is also the only locality that that exhibits the unique pattern of RCD only in pulse number, compared to other localities that diverged in pulse rate or in both variables (Lemmon, 2009).

### 4.5 Evidence for reinforcement driving prezygotic isolation

In this study, we provide evidence to support the last of the five criteria proposed by Howard (1993) to show that reproductive character displacement was driven by reinforcement. Previous work indicated the following: (1) strong selection against hybridization (Lemmon & Lemmon, 2010), (2) divergence in male mating signals is perceptible to females (Lemmon, 2009), (3) the reproductive signal is heritable (Lemmon, 2009), and (4) reproductive character divergence is not driven by other factors, particularly ecology (Malone, Ribado, J., & Lemmon, E. M. 2014). Here, we provide support for the final criterion, showing that hybridization is widespread but variable in degree across the entire contact zone. Thus, the chorus frog system represents a well‐supported empirical example of how reinforcement can lead to the evolution of both female preferences and male signals, resulting in enhanced prezygotic isolation. Furthermore, observed patterns of genetic differentiation are consistent with cascade reinforcement, through which interactions between species can have the indirect effect of accelerating divergence within species. Future work will focus on understanding the costs of hybridization for *P. nigrita* and the consequences of interactions between *P. feriarum* and other closely related taxa for diversification with species.

## AUTHOR CONTRIBUTIONS

EML and TJ developed the conceptual framework for the project, EML collected and analyzed the data, and EML and TJ both contributed to writing the manuscript.

## DATA ARCHIVAL LOCATION

Dryad Data Repository (www.datadryad.com).

## CONFLICT OF INTEREST

None declared.
